# A study of two Chinese patients with tetrasomy and pentasomy 15q11q13 including Prader-Willi/Angelman syndrome critical region present with developmental delays and mental impairment

**DOI:** 10.1186/1471-2350-14-9

**Published:** 2013-01-15

**Authors:** Jing Yang, Yongchen Yang, Yi Huang, Yan Hu, Xi Chen, Hengjuan Sun, Zhibao Lv, Qian Cheng, Liming Bao

**Affiliations:** 1 Center for Clinical Molecular Medicine; Ministry of Education Key Laboratory of Child Development and Disorders; Key Laboratory of Pediatrics in Chongqing; Chongqing International Science and Technology Cooperation Center for Child Development and Disorders, Children's Hospital of Chongqing Medical University, Chongqing, China; 2 Shanghai Children's Hospital, Shanghai Children's Hospital Affiliated to Shanghai Jiao Tong University School of Medicine, Shanghai, China; 3 Department of Child Health Care and Growth Developmental and Psychological Health Center, Children's Hospital of Chongqing Medical University, Chongqing, China; 4 Division of Human Genetics, Department of Pediatrics, Cincinnati Children’s Hospital Medical Center and University of Cincinnati College of Medicine, Cincinnati, OH, USA

**Keywords:** Chromosome 15, Cytogenomic array, Copy number, Pentasomy, Tetrasomy

## Abstract

**Background:**

The proximal chromosome 15q is prone to unequal crossover, leading to rearrangements. Although 15q11q13 duplications are common in patients with developmental delays and mental impairment, 15q aneusomies resulting in greater or equal to 4 copies of 15q11q13 are rare and no pentasomy 15q11q13 has been reported in the literature. Thus far, all reported high copy number 15q11q13 cases are from the West populations and no such study in Chinese patients have been documented. Dosage-response pattern of high copy number 15q11q13 on clinical presentations is still a subject for further study.

**Case Presentation:**

In this study, we characterized two Han Chinese patients with high copy number 15q11q13. Using chromosome banding, high resolution SNP-based cytogenomic array, Fluorescence *in situ* hybridization, and PCR-based microsatellite analysis, we identified two patients with tetrasomy 15q11q13 and pentasomy 15q11q13. Both 15q11q13 aneusomies resulted from a maternally inherited supernumerary marker chromosome 15, and each was composed of two different sized 15q11q13 segments covering the Prader-Willi/Angelman critical region: one being about 10 Mb with breakpoints at BP1 and BP5 regions on 15q11 and 15q13, respectively, and another about 8 Mb in size with breakpoints at BP1 and BP4 regions on 15q. Both patients presented with similar clinical features that included neurodevelopmental delays, mental impairment, speech and autistic behavior, and mild dysmorphism. The patient with pentasomy 15q11q13 was more severely affected than the patient with tetrasomy 15q11q13. Low birth weight was noted in patient with pentasomy 15q1q13.

**Conclusions:**

To the best of our knowledge, this is the first case of pentasomy 15q11q13 and the first study of high copy number 15q11q13 in Han Chinese patients. Our findings demonstrate that patients with tetrasomy and pentasomy of chromosome 15q11q13 share similar spectrum of phenotypes reported in other high copy number 15q11q13 patients in the West, and positive correlation between 15q11q13 copy number and degree of severity of clinical phenotypes. Low birth weight observed in the pentasomy 15q11q13 patient was not reported in other patients with high copy number 15q11q13. Additional studies would be necessary to further characterize high copy number 15q11q13 aneusomies.

## Background

The proximal long arm of chromosome 15 (15q) contains repeat sequences that are prone to misalignments and unequal crossover during meiosis, leading to deletions, duplications, and supernumerary chromosomes [1-4]. Deletions of 15q11q13 involving the Prader-Willi/Angelman syndrome critical region (PWACR) may cause Prader-Willi syndrome [MIM 176270] and Angelman syndrome [MIM 105830], depending on the parental origin [1]. Duplications of 15q11q13, particularly of maternal origin, are associated with clinical features, including psychomotor delays, cognitive disability, epilepsy, growth retardation, and behavioral problems [3,5]. Derivative chromosomes 15q, mostly in a form of inverted duplication of 15q (inv dup(15q)), are the most common supernumerary marker chromosomes (SMC) in humans [2,6,7]. Because of their clinical relevance, SMC15s spanning PWACR have been the focus of studies. Clinical features associated with tetrasomy 15q11q13 have been reported but the underlying molecular characteristics have not been well characterized [2]. In contrast, aneusomies resulting in high copy number (≥ 4 copies) 15q11q13 segment, particularly greater than four copies, are rare. So far, only a dozen of hexasomy 15q11q13 cases have been reported in the literature and none were pentasomy 15q11q13 [4,8]. All of the patients with high copy number 15q11q13 reported thus far are from the West populations.

Most high copy number 15q11q13 aneusomies were studied by chromosome banding and fluorescence *in situ* hybridization (FISH), only few were analyzed at molecular levels. Recent advances in high density cytogenomic arrays provide powerful tools in detecting submicroscopic copy number change and delineating chromosome breakpoints. Here, we report a study of two Han Chinese patients with tetrasomy and pentasomy 15q11q13 who present with developmental delays and cognitive disabilities.

## Case presentation

### Clinical data

**Figure 1:**
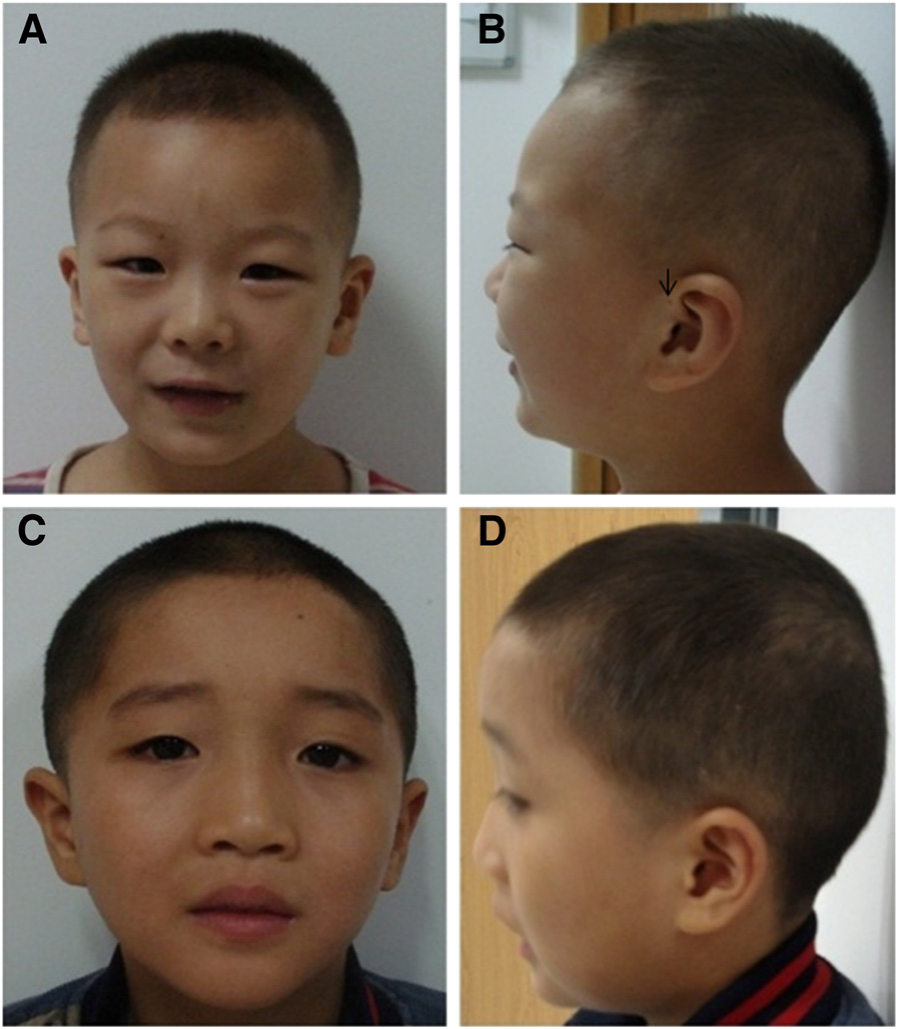
**Patients photographs.** Frontal (**A**) and side (**B**) views of patient 1 showing upper thin lip, preauricular fistula (arrowed) and hypertelorism. Frontal (**C**) and side (**D**) views of patient 2 showing hypertelorism, slightly anteverted nares and low-set ears.

Patients described in this study were enrolled in a large study of genomic aberrations in patients with developmental delays and intellectual disabilities at the Children’s Hospital of Chongqing Medical University, Chongqing, China. The study was approved by the hospital’s Ethics Committee. Patient 1 was a 5-year-old boy who had a history of developmental delays, intellectual disabilities, speech delay, and behavioral problems. He was the child of a healthy non-consanguineous couple. Family history was unremarkable. The gravida 1 para 1 mother was 25, and the father, 28, at the time of his birth. The mother had no history of miscarriages, and the pregnancy was uneventful. The patient was delivered at full-term, birth weight was 1,800 g (3^th^ percentile); length, 47 cm (25^th^ percentile); occipital frontal circumference, 32 cm (25^th^ percentile). Resuscitation was performed at birth because of suffocation of unknown cause. No signs of asphyxia, jaundice, feeding problems, infections, or other problems were reported in the post-natal follow-up. Head control was achieved at 3 months; standing with aid at 1 year; and walking at 2 years. He started saying simple words at 2 years, and his speech was monosyllabic at 5. Behavioral problems started at 2. Evaluation at age of 5 showed that the patient was aggressive, short tempered, and had a tendency toward outbursts and being anger. He was hyperactive, impulsive, failed to follow instructions and rules, and was destructive. He had difficulties in focusing and was not able to finish tasks. He was easy to get into fights with peers, and did not do well in group activities. Physical exams showed slight microcephaly, thin upper lip, preauricular fistula, hypertelorism (Figures 1A and 1B), moderate hypotonia at low extremities, and unable to stand on one foot and to run. Ligamentous laxity at the ankle joint was noted. At the age of 5, according to the scale of Gross Motor Function Measure [9], his scores for lying and rolling were 97; crawling and kneeling, 77; sitting, 100; standing, 79; and walking, 67. Based on Gesell Developmental Observation [10] (5 years old) his social adaptive skills were at 20 months; organizational skills, 15 months; motor skills, 18 months; and language skills, 19 months. Electroencephalography (EEG) study and cranial CT were normal, and no cardiac defects were detected. Patient 2 was born to a 34-year-old mother and 29-year-old father who are not related. The pregnancy and delivery were uneventful. His birth weight was 3,700 g (75^th^ percentile); length, 55 cm (50^th^ percentile); head circumference, 35 cm (50^th^ percentile). He did not walk until 2 years old, said simple words at 3, and started to play with peers at 4. At age of 8 years, he had difficulties in standing straight on one foot and had joint laxity. He showed hypertelorism, slightly anteverted nares, and low-set ears (Figures 1C and 1D). He had pathic facial expression and said simple sentences. By age of 9 years, his body weigh was 31.5 kg (95^th^ percentile); height, 125 cm (50^th^ percentile); and head circumference, 53 cm (75^th^ percentile). He had no problems in walking and running. He spoke simple sentences and was not able to repeat stories. The behavioral problems started at age of 2 years. He did not like to join group activities, and had tendency to be aggressive and destructive. Evaluations with the Weshsler Intelligence Scale for Children, fourth edition (WISC-IV) [11] showed his four index cores scores at age of 9 were: verbal comprehension, 45; perceptual reasoning, 40; working memory, 50; processing speed, 46 and full scale IQ, 41. EEG study and cranial CT were all normal.

### Genetic analysis

**Figure 2:**
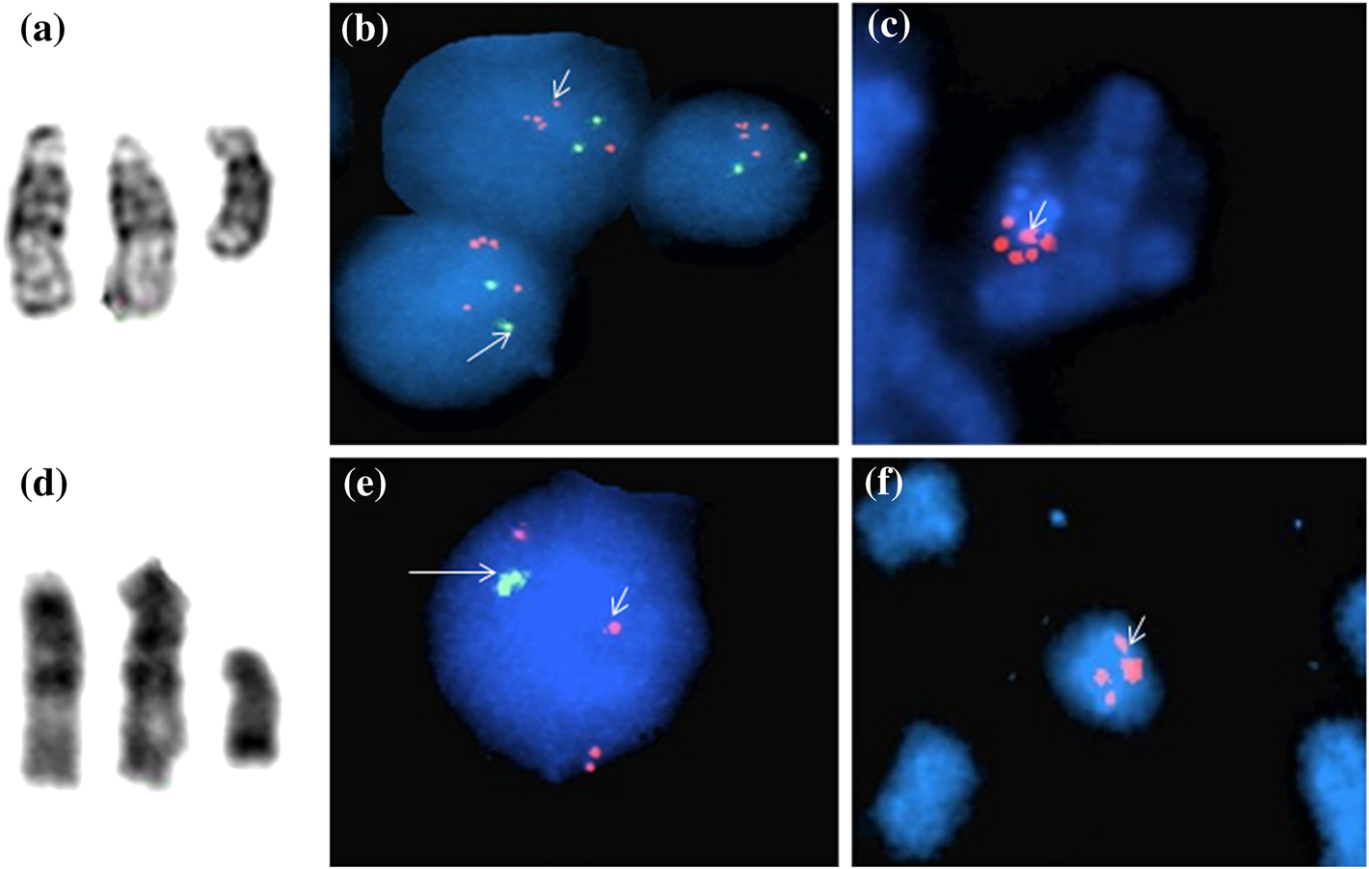
**Partial karyotype and FISH analysis.** Patient 1: (**a**) partial karyotype of derivative chromosome 15. FISH study using probes for *SNRPN* locus (orange, short arrows) at 15q11q13 and *PML* locus (green, long arrows) at 15q22: (**b**) interphase cells and (**c**) SMC15. Patient 2: (**d**) partial karyotype of inv dup(15), FISH using *SNRPN* and *PML* probes: (**e**) interphase cells and (**f**) inv dup(15q).

**Figure 3:**
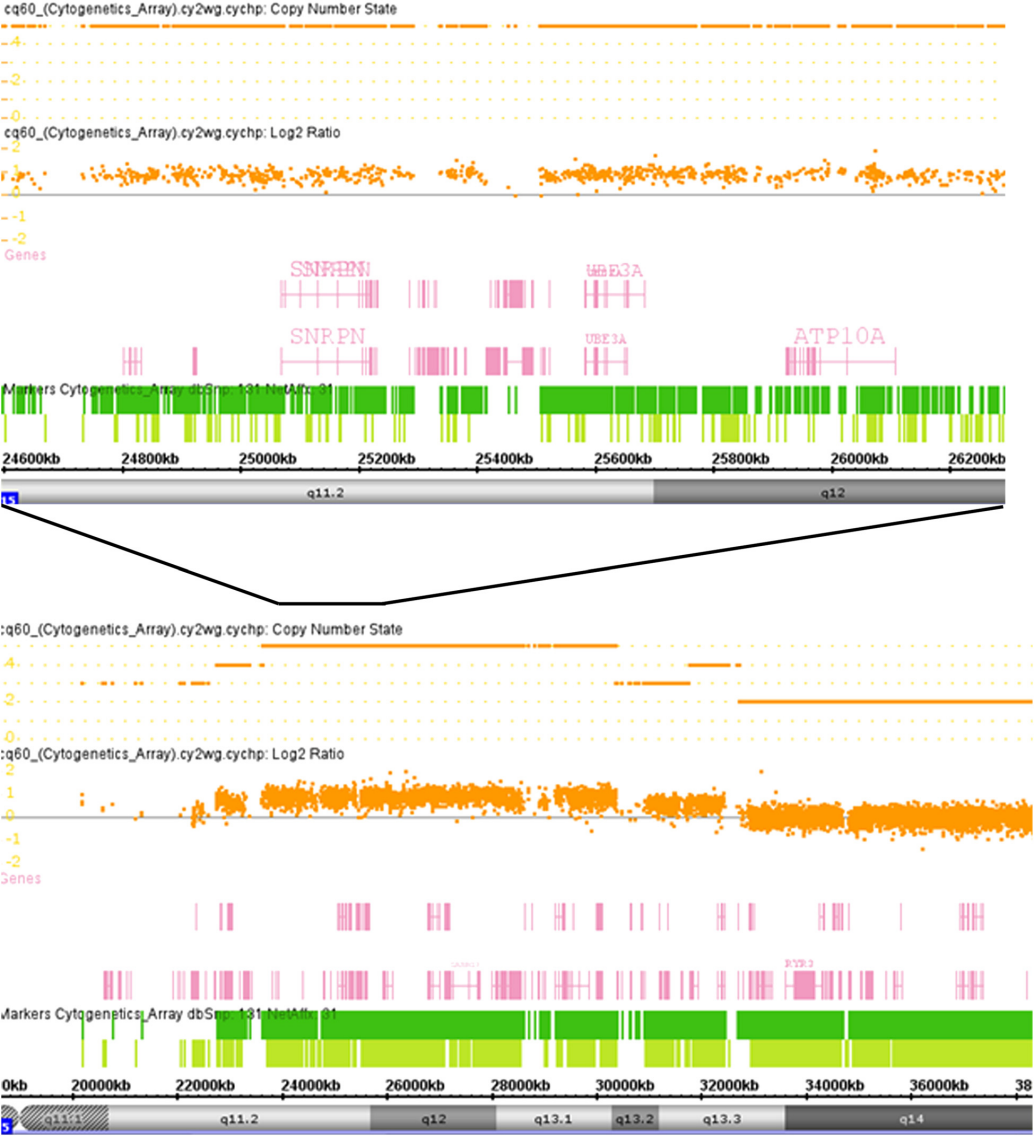
**Cytogenomic array analysis showing copy number for chromosome 15q11q13 segment in patient 1.** The lower portion of the figure shows array data from proximal chromosome 15q, and the upper portion represents the enlarged part of affected 15q11q13.

**Figure 4:**
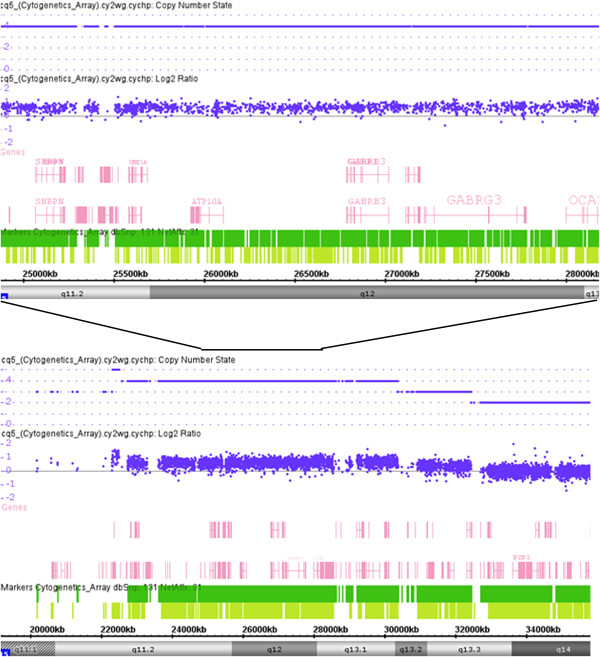
**Cytogenomic array analysis of patient 2 showing copy number change on chromosome 15q11q13.** The lower portion of the figure shows array data from proximal chromosome 15q, and the upper portion represents the enlarged part of affected 15q11q13.

Banded chromosome analysis of peripheral blood revealed each patient had a SMC15: a rea(15)(q11) or 47,XY,+rea(15)(q11) in patient 1 and an inv dup(15)(q11q13) or 47,XY,+inv dup(15)(q11q13) in patient 2 (Figure 2). Parental cytogenetic studies were normal. Genomic DNA from the patients was prepared and hybridized to SNP-based Cytoscan HD array (Affymetrix, USA). The cytogenomic arrays had 2.69 million markers covering the entire human genome with the average spatial distance between intragenic neighboring markers of 880 bases. Cel intensity or CEL files were created using Affymetrix GeneChip Command Console operating software and Chromosome Analysis Suite, according to manufacturer’s protocols. Copy number changes were detected using a Hidden Markov Model algorithm [12]. Gene annotations were determined using build GRCh37/hg19. The array analysis revealed increases in 15q11q13 copy number in both patients. Patient 1 had 5 copies of 15q11.2q13.2 (7.72 Mb, chr15:22,619,671-30,342,457), and 3 copies of 15q13.2q13.3 (2.89 Mb, chr15:30,342,458-32,411,629), indicating the SMC15 was comprised of 2 copies of 15q11.2q13.2 (7.72 Mb, chr15:22,619,671-30,342,457) and 1 copy of 15q11.2q13.3 (10.61 Mb, chr15:22,619,671-32,411,629) (Figure 3). Patient 2 had 4 copies of 15q11.2q13.1 (8.00 Mb, chr15:22,054,840-30,058,502) and 3 copies of 15q13.1q13.2 (2.36 Mb, chr15:30,058,503-32,418,417), demonstrating that the inv dup(15q) contained one 8.00-Mb 15q11.2q13.1 segment (chr15:22,054,840-30,058,502) and another 10.36-Mb 15q11.2q13.3 segment (chr15:22,054,840-32,418,417) (Figure 4). FISH using the *SNRPN* probe (Abbott Molecular, USA) showed that in addition to 2 signals on normal chromosome 15 homologues, the SMC15 from patient 1 had 3 signals (Figures 2b and 2c), and the inv dup(15q) from patient 2 had 2 signals (Figures 2e and 2f), confirming the array findings of pentasomy and tetrasomy 15q11q13 in patients 1 and 2, respectively.

Parental origin of 15q11q13 aneusomies was analyzed by PCR-based genotyping of 12 STS loci on 15q11q13. Informative markers in patient 1, *D15S975*, *D15S156R*, *D15S1043* and *D15S123*, showed 1 and 2 copies of paternal and maternal alleles, respectively, consistent with maternal inheritance of the SMC15. Informative alleles in patient 2, *D15S817*, *D15S97* and *D15S1048*, were 1 and 2 copies of paternal and maternal alleles, respectively, also suggesting maternal origin of the inv dup(15) (Table 1). Quantitative PCR methylation analysis of *SNRPN* was performed to determine methylation pattern of 15q11q13. Patient genomic DNA was pre-treated with bisulfite using EZ DNA Methylation-Gold kit (Zymo, USA). Primers for methylated and unmethylated *SNRPN* were 5^′^-GAGGGAGTTGGGATTTTTGTATTG-3^′^ and 5^′^-CCCAAACTATCTCTTAAAAAAAACCAC-3^′^, and 5^′^-CTCCAAAACAAAAAACTTTA AAACCCAAATTCC-3^′^ and 5^′^-GTGAGTTTGGTGTAGAGTGGAGTGGTTGTTG-3^′^, respectively. The PCR conditions were 1 cycle at 95°C for 2 min, and 45 cycles at 95°C for 15 s, 61°C for 30 s, and 72°C for 45 s. Each reaction was done in triplicate. The β-actin gene was used as an internal control. Both patients showed 1 copy of unmethylated (paternal pattern) *SNRPN*, and 4 and 3 copies of methylated (maternal pattern) *SNRPN* in patients 1 and 2, respectively (data not shown), demonstrating maternal imprinting pattern of both SMC15s.

**Table 1:** Parental origin analysis of derivative chromosomes 15

	**Patient 1**			**Patient 2**		
STS	Father	Mother	Proband	Father	Mother	Proband
*D15S541*	1,2	1,2	1,2	1,1	1,2	1,2
*D15S817*	1,1	2,2	2,2	**1,1**	**2,3**	**1,2,3**
*GABR3*	1,1	1,1	1,1	1,1	1,1	1,1
*D15S97*	1,2	1,2	1,2	**1,2**	**3,4**	**1,3,4**
*D15S975*	**1,2**	**3,4**	**1,3,4**	1,2	1,2	1,2
*D15S156R*	**1,2**	**3,4**	**1,3,4**	1,2	1,3	1,3
*D15S815*	1,1	1,1	1,1	1,2	1,3	1,3
*D15S1048*	1,2	1,2	1,2	**1,1**	**2,3**	**1,2,3**
*D15S1043*	**1,1**	**2,3**	**1,2,3**	1,2,	1,3	1,3
*D15S1013*	1,2	1,1	1,2	1,1	2,2	1,2
*D15S123*	**1,1**	**2,3**	**1,2,3**	1,2	1,2	1,2
*D15S1007*	1,2	1,2	1,1	1,2	1,2	1,2

## Discussion

Here, we report tetrasomy and pentasomy 15q11q13 in two Han Chinese patients. To the best of our knowledge, pentasomy 15q11q13 has not been reported in the literature and the work presents the results of the first study of high copy number 15q11q13 in Chinese patients. Most clinical features in our patients including neurodevelopmental delays, intellectual disabilities, speech problems, and mild dysmorphisms overlap with the ones previously described in high copy number 15q11q13 patients in the West [2,4,8,13]. However, some differences were noted. Seizures are present in two-thirds of tetrasomy patients with the age of onset ranging from 6 months to 9 years [2,8], and were also reported in ten patients with hexasomy 15q11q13 starting at less than 3 years old. Seizures had not been developed in our patients with tetrasomy and pentasomy 15q11q13 at age of 5 and 9 years, respectively. Vision deficits are uncommon in patients with trisomy and tetrasomy 15q11q13, and were seen in 3 of 10 hexasomy patients [2,8,14]. Neither of the patients in this study had vision deficits. Severe developmental delays and intellectual disabilities are prominent features in hexasomy 15q11q13, and the pentasomy patient reported here also showed profound developmental delays and cognitive deficits. Of 8 hexasomy patients whose language development was evaluated, 6 had absent or nonverbal speech and 2 was poor speech [4,8]. The pentasomy 15q11q13 patient had severe speech problems and was only able to say simple words at 5. The tetrasomy patient said simple sentences at 9. Although both patients reported in this study demonstrated behavioral problems overlapping with some features in patients with autism, the severity of psychomotor retardation in the patients was incompatible with a diagnosis of autism. Another notable feature in patient with pentasomy 15q11q13 is low birth weight which was not reported in other patients with high copy number 15q11q13 [8,15,16]. Low birth weight might contribute to development delays. Clinical presentations observed in the patient with tetrasomy 15q11q13 overlap those described in Western patients with similar 15q aneusomy [2]. Although both tetrasomy and pentasomy patients shared similar clinical profiles, pentasomy patient appeared to be more severely affected than tetrasomy patient with regard to development delays and speech problems. These observations suggest that the increase in copy number in 15q11q13 aneusomies may not change clinical features, but result in increased severity. This is in agreement with previous reports from the studies of patients with no more than four copies of 15q11q13 [2,4,8].

Clinical heterogeneity among patients with various 15q11q13 aneusomies may be attributed to differences in copy number, the extent of affected regions, parental origin, age at evaluation, available clinical information, and the presence of mosaicism. Study of additional cases with high copy number 15q11q13 would be necessary for a better understanding of clinical phenotypes associated with high copy number 15q11q13.

The pentasomy and tetrasomy 15q11q13 reported here were all materially inherited. Genes in the BP1–BP4 region that are maternally imprinted and paternally expressed, including *MKRN3, NDN, MAGEL2* and *SNURF*/*SNRPN*, are unlikely to contribute to the clinical phenotypes. However, genes that are maternally preferentially expressed in the brain, such as *UBE3A* and *ATP10A*, may increase in expression and have clinical effects. Other genes in the region, including those for GABA_A_ receptor subunits (*GABRB3, GABRA5,* and *GABRG3*) and *ABPA2* are not subject to imprinting and are biallelically expressed. The GABA_A_ receptor genes have been implicated in autism, and *ABPA2* plays a critical role in synaptic vesicle exocytosis in the brain [17,18]. The *CHRFAM7A* and *CHRNA7* genes that reside in the BP4–BP5 region have been linked to psychiatric disorders [19].

Most high copy number 15q11q13 abnormalities were studied by chromosome banding and/or FISH analysis, and few were characterized at molecular levels. Using high-resolution cytogenomic array, we were able to delineate high copy number 15q11q13 aneusomies at high precision (average spatial distance between intragenic neighboring markers: 880 bases). The breakpoints for the large 15q11q13 segments in both SMC15s fall into the same BP1 and BP5 regions on proximal 15q, while the breakpoints for small 15q11q13 segments were at BP1 and BP4 regions. The PWACR region (4 Mb) is located between BP2 and BP3. Our results concur with the findings from previous studies that distal breakpoints for large 15q11q13 rearrangements often fall within BP4 or BP5 region and BP4 is more prone than BP5 to recombination [20-22].

## Conclusions

We identified two Han Chinese patients with tetrasomy and pentasomy 15q11q13 containing PWACR. To the best of our knowledge, this is the first pentasomy 15q11q13 case and the first study of high copy number 15q11q13 in Chinese populations. Both 15q11q13 aneusomies were maternally inherited and composed of two sized 15q11q13 segments with similar proximal breakpoints at BP1 and distal breakpoints at BP4/BP5. The patients shared similar spectrum of clinical features including neurodevelopmental delays, intellectual disabilities, speech delay, autistic behaviors, and mild dysmorphism, with pentasomy patient being more severely affected than tetrasomy patient. Low birth weight observed in the pentasomy patient has not been previously described in patients with high copy number 15q11q13. Our findings suggest positive relationship between 15q11q13 copy number and degree of severity of clinical presentations.

### Consent

Written informed consents were obtained from patient parents for publication and the accompanying images. Copies of the written consents are available for review by the Editor-in-Chief of this journal.

## Abbreviations

PWACR: Prader-Willi/Angelman syndrome critical region;SMC: Supernumerary marker chromosome;PCR: Polymerase chain reaction;BP: Breakpoint

## Competing interests

The authors declaim no competing interests.

## Authors’ contributions

LB, QC and YH wrote the manuscript; LB, QC, YY, YH, and ZL participated in study design, data interpretation; JY, YH QC, XC, YH and HS collected data; JY, YH, YH, XC, YY and HS conducted experiments and data analysis. All authors read and approved the final manuscript.

## Pre-publication history

The pre-publication history for this paper can be accessed here:

http://www.biomedcentral.com/1471-2350/14/9/prepub
